# Retraction Note: Effects of miR-672 on the angiogenesis of adipose-derived mesenchymal stem cells during bone regeneration

**DOI:** 10.1186/s13287-022-03226-y

**Published:** 2023-01-12

**Authors:** Mingjiao Chen, Meng Zhou, Yao Fu, Jin Li, Zi Wang

**Affiliations:** Shanghai Key Laboratory of Orbital Diseases and Ocular Oncology, Department of Ophthalmology, Ninth People’s Hospital, Shanghai Jiao Tong University School of Medicine, Zhizaoju Road No. 639, Shanghai, 200011 People’s Republic of China

**Retraction Note: Stem Cell Research & Therapy (2021) 12:85** https://doi.org/10.1186/s13287-021-02154-7

The authors have retracted this article. After publication the authors noted that they used the incorrect β-actin protein expression data in Fig. 1E. Yao Fu did not participate in the experiments presented and were included in the author list without their permission. Meng Zhou, Yao Fu and Zi Wang agree to this retraction. Mingjiao Chen and Jin Li do not agree to this retraction.

